# Secretome from human adipose-derived mesenchymal stem cells promotes blood vessel formation and pericyte coverage in experimental skin repair

**DOI:** 10.1371/journal.pone.0277863

**Published:** 2022-12-19

**Authors:** Brysa M. Silveira, Tiago O. Ribeiro, Railane S. Freitas, Ana C. O. Carreira, Marilda Souza Gonçalves, Mari Sogayar, Roberto Meyer, Alexander Birbrair, Vitor Fortuna

**Affiliations:** 1 Health Science Institute, Federal University of Bahia, Salvador, BA, Brazil; 2 Department of Surgery, School of Veterinary Medicine and Animal Science, University of São Paulo, São Paulo, Brazil; 3 Gonçalo Moniz Institute, Oswaldo Cruz Foundation, Salvador, BA, Brazil; 4 Department of Clinical Analysis, Faculty of Pharmacy, Federal University of Bahia, Salvador, BA, Brazil; 5 Cell and Molecular Therapy Center (NUCEL), Medical School, University of São Paulo, São Paulo, Brazil; 6 Biochemistry Department, Chemistry Institute, University of São Paulo, São Paulo, SP, Brazil; 7 Department of Pathology, Federal University of Minas Gerais, Belo Horizonte, MG, Brazil; 8 Department of Radiology, Columbia University Medical Center, New York, NY, United States of America; 9 Department of Dermatology, School of Medicine and Public Health, University of Wisconsin-Madison, Madison, Wisconsin, United States of America; TotiCell Limited, Bangladesh, BANGLADESH

## Abstract

Human adipose tissue-derived stem cells (hASC) secretome display various therapeutically relevant effects in regenerative medicine, such as induction of angiogenesis and tissue repair. The benefits of hASC secretome are primarily orchestrated by trophic factors that mediate autocrine and paracrine effects in host cells. However, the composition and the innate characteristics of hASC secretome can be highly variable depending on the culture conditions. Here, we evaluated the combined effect of serum-free media and hypoxia preconditioning on the hASCs secretome composition and biological effects on angiogenesis and wound healing. The hASCs were cultured in serum-free media under normoxic (NCM) or hypoxic (HCM) preconditioning. The proteomic profile showed that pro- and anti-antiangiogenic factors were detected in NCM and HCM secretomes. *In vitro* studies demonstrated that hASCs secretomes enhanced endothelial proliferation, survival, migration, *in vitro* tube formation, and *in vivo* Matrigel plug angiogenesis. In a full-thickness skin-wound mouse model, injection of either NCM or HCM significantly accelerated the wound healing. Finally, hASC secretomes were potent in increasing endothelial density and vascular coverage of resident pericytes expressing NG2 and nestin to the lesion site, potentially contributing to blood vessel maturation. Overall, our data suggest that serum-free media or hypoxic preconditioning enhances the vascular regenerative effects of hASC secretome in a preclinical wound healing model.

## Introduction

Human adipose tissue-derived stem cells (hASCs) are a promising therapeutic strategy in regenerative medicine explored in various models of tissue repair, ischemic injuries, immune disorders [1, 2], also to improve chronic non-healing or recurrent cutaneous wounds, such as age-associated delayed healing, chronic diabetic wounds, and irradiated wounds [3, 4]. Many preclinical and clinical studies have demonstrated that the hASCs secretome enhances wound healing and vascularization mainly by stimulating cell survival, angiogenesis, cell proliferation, ECM cell migration/adhesion, and reducing inflammation [5, 6]. Administration of the hASC’s secretome to injured skin also increases its local metabolic activity, oxygen supply, and extracellular matrix remodeling [7], accelerating tissue healing.

The beneficial effects of *in vivo* hASCs are primarily orchestrated by their secretome enriched with trophic molecules (represented by cytokines and growth factors) and a vesicular fraction (microvesicles and exosomes) that mediate autocrine and paracrine cell communication [8, 9]. Therefore, identifying critical factors secreted and characterization of their functional roles in cutaneous wound healing can be a practical approach to designing more potent secretome-based therapeutics with more predictable clinical outcomes [4]. Recently, proteomic analysis of hASCs secretome has found many players that improve wound healing *in vivo*, including epidermal growth factor (EGF), fibroblast growth factor-2 (FGF2), hepatocyte growth factor (HGF), fibroblast growth factor (FGF), platelet-derived growth factors (PDGFs), insulin-like growth factor (IGF)-1 and 2, and stromal cell-derived factor [4, 5, 10]. Kim and colleagues [11] identified MCP-1, IL8, VEGF, and angiogenin as efficient secretome biomarkers for predicting vascular regenerative efficacy in wound healing [11]. MicroRNAs that could be taken up by endothelial cells and stimulate *in vitro* and i*n vivo* sprouting angiogenesis were also described in hASC secretome [12–15].

However, the hASC secretome’s composition and innate characteristics can vary depending on the cell source and culture conditions. Hypoxic preconditioning is one of the most frequent ways to improve hASC secretome, as hypoxic stress reduces oxygen, improves cellular function, and increases the concentration of paracrine factors [16, 17] that promotes wound healing [18–21] with fewer scar formation [22–24] in comparison to normoxic conditions. Furthermore, hASCs secretome produced in serum-free media has enhanced immunosuppressive and anti-fibrotic abilities because of increased vascular endothelial growth factor (VEGF) and hepatocyte growth factor (HGF) secretion [25, 26]. These findings led us to the hypothesis that serum-free media and hypoxic preconditioning would synergistically enhance the therapeutic effects of hASCs secretome on wound healing.

In this study, first, we characterized cultivated hASCs and the soluble angiogenic-related and tissue repair factors secreted in serum-free media under 1% O_2_ (hypoxic) (HCM) or normoxic conditions (NCM). Next, we assessed the secretomes’ angiogenic and tissue regeneration potential using HUVECs primary culture. We demonstrated that the hASCs secretome activated the PI3K/Akt signaling cascade and enhanced endothelial proliferation, survival, migration, and *in vitro* tube formation. A Matrigel plug assay showed that NCM and HCM were potent promoters of *in vivo* angiogenesis. Next, using a pericyte labeled (or reporter) transgenic nestin‐GFP/NG2‐DsRed mice revealed that hASC secretomes accelerated wound healing, increased endothelial density, and vascular coverage with resident pericytes expressing NG2 and nestin to the lesion site, potentially contributing to blood vessel maturation. Overall, our data suggest that serum-free media or hypoxic preconditioning enhances hASCs secretome’s vascular regenerative effects by directly recruiting NG2+nestin+ pericytes to the injury site.

## Material and methods

### Ethics statement

This study was approved by the institutional review board of the Health Science Institute (Federal University of Bahia, approval no 2.074.627). This study is in compliance with the ethical principles of the revised Declaration of Helsinki. All participants read, understood and gave written consent in the form approved by the institutional review board before agree to the study activities.

### Animals

All animal experiments were reviewed, approved and performed in accordance with Brazilian guidelines and regulations. The institutional review board approved animal handling and procedures for animal experimentation (CEUA, UFBA-2018-131). All animal procedures were carried out in strict accordance with the Guide for the Care and Use of Laboratory Animals and the regulation of animal protection committee to minimize the suffering and injury. The animal studies are in compliance with the Animal Research: Reporting of In Vivo Experiments (ARRIVE) guidelines.

C57BL/6 mice and Nestin-GFP+/NG2-DsRed+ double-transgenic mice colonies were housed at the Health Science Institute of the Federal University of Bahia in a pathogen-free facility under a 12-hour:12-hour light/dark cycle, with *ad libitum feeding*. Nestin-GFP+/NG2-DsRed+ double-transgenic mice have been described [27]. In brief, they are transgenic mice expressing GFP under the nestin promoter and DsRed+ under the NG2 promoter. Our Nestin-GFP+/NG2-DsRed+ double-transgenic mice colony was maintained homozygous for the transgenes on the C57BL/6 genetic background. Both male and female homozygous mice were used, and their ages ranged from eight to twelve-week-old. The mice were monitored daily and euthanized humanely by overdose (three times the dose used to anesthetize) of ketamine plus xylazine (80 and 20 mg kg^−1^ i.p.) at the end of the experiment or the first sign of shortness of breath, reduced locomotion and reduced body weight.

### Human cell culture

The hASC were isolated from lipoaspirate harvested as surgical waste products [28]. In order to avoid gender-related variability, only female donors were selected for this study. Following well-established isolation procedures, hASC populations were obtained from 8 healthy women (S1 Table) (aged 25–45 years old). A volume of 50 mL lipoaspirate was treated with collagenase I (1 mg/mL) (Sigma, C0130-1G) at 37°C, and 30 min later, the digested material was centrifuged to obtain the stromal vascular fraction (SVF). The SVF was cultured in DMEM with low glucose (Dulbecco’s Modified Eagle’s Medium, Life Technologies), supplemented with 10% FBS, antibiotics (PenStrep and gentamicin) in adherent culture bottles. hASCs were expanded in a standard culture medium in a humidified atmosphere at 37°C with routine passaging at 80% confluence. The culture medium was renewed every three days. hASCs at passages three to six were used for all subsequent experimentation.

Primary human umbilical vein endothelial cells (HUVECs) cells were isolated from umbilical cords as described [29]. The HUVECs were cultured in EGM2/BulletKit medium (Lonza Group Ltd.) supplemented with 100 U/mL penicillin/streptomycin (Life Technologies) at 37°C in 5% CO_2_ and 95% air. HUVECs were seeded on 0.1% gelatin (Sigma), and EGM2/BulletKit was replaced every 2–3 days. HUVECs at passages three to six were used for this study.

### Immunophenotypic and multipotency characterization of hASCs

The expression of cell-surface antigens using fluorescein isothiocyanate (FITC)-conjugated and phycoerythrin (PE)-conjugated antibodies was examined in hASCs (1 x 10^5^ cells) using the following antibodies: anti-CD14 PE (clone 61D3, Lot: E026669, ebioscience), anti-CD34FITC (BD, Cat No. 348053), anti-CD45PerCP (lot: ED7029, Exbio), anti-CD146 PE (lot: 91811, BD Biosciences), anti-HLA-DR PE (clone: MEM-12, lot: 1P474T100, Exbio), antiCD29FITC (clone: TS2116, lot: E031567, ebioscience), anti-CD73 PE (clone: AD2, lot: 1P675T100, exbio), anti-CD90 (clone: ebioE10, lot: E0228253, ebioscience) PE, anti-CD105FITC (clone: SN6, lot: E029268, ebioscience) and appropriate isotype control antibodies from the same manufacturers. Samples were run on a FACScalibur flow cytometer (BD Biosciences, CA, USA) and analyzed using a BD Cell Quest pro software.

The multipotency capacity of hASC for trilineage differentiation (osteogenic, chondrogenic, and adipogenic) and the intracellular expression of mesenchymal markers were assessed as described [30, 31]. Immunofluorescence staining was carried out on hASCs (2.0 x 10^4^ cells/glass coverslips) with the following primary antibodies: mouse anti-SMA (Sigma, 1:500 in BSA/standard serum solution), and rabbit anti-Collagen IV (Abcam, 1:100). After PBS washing, cells were incubated with the following secondary antibodies: Alexa Fluor-555 anti-mouse; Alexa Fluor-488 anti-rabbit (Molecular Probes, 1:500 in BSA/standard serum solution).

### Conditioned Medium preparation and collection

Conditioned Medium (CM) preparation was performed as described previously [30], with some modifications. The CM was obtained after 1 × 10^6^ cell hASCs (passages 3–6) were cultured for 48h with 3 mL of serum-free EBM^TM^-2 Basal Medium (Lonza), supplemented with 1% bovine serum albumin (Sigma-Aldrich Co) in T25 culture flasks either under standard conditions (normoxic, NCM) or combined with 0.5% of oxygen preconditioning (hypoxic, HCM) [30]. Hypoxic preconditioning was produced in an Anaerobac Jar (Probac, São Paulo, Brazil) for 48h [32]. CMs were then collected, centrifuged at 3,000 rpm for 20 min at 4°C to remove cell debris and large apoptotic bodies, and maintained at -70°C until use. The media collected were referred to as normoxic hASC conditioned medium (NCM) or hypoxic hASC conditioned medium (HCM), respectively. In order to obtain results that were not affected by single donor variability and more representative of trophic factors released by hASCs, we pooled CMs from 2 different hASCs at the same passage, and used these pools for each specific analysis. Total protein content of conditioned medium samples was quantified using the Bradford Protein Assay according to manufacturer’s protocol. Bovine serum albumin standards were used. The total protein concentration of the CM pools was normalized based on the average total protein content (2.50 +/- 0.15 mg/mL) achieved after 48h of incubation of the confluent hASC monolayer. Identical replicate doses of these normalized CMs were used in all the experiments.

### Electrophoresis and Western blot analysis

HUVEC cell lysates were prepared in sodium dodecyl sulfate (SDS) buffer containing Complete Mini Proteinase Inhibitor Cocktail Tablets (Sigma-Aldrich®). Samples (100μg) of the protein lysates were loaded onto 12% polyacrylamide SDS gel, and the separated proteins were then transferred to polyvinylidene difluoride (PVDF) membranes. After transfer, the membrane was blocked and incubated overnight with the following primary antibodies: β-Actin, AKT, and phospho-AKT (Ser473) (Cell Signaling Technology). The membrane was washed and incubated for one hour with a secondary antibody (Peroxidase Goat Anti-Rabbit IgG Antibody PI1000, Vector Laboratories). For development, the membrane was immersed in a chemiluminescent solution (Immobilon Western Chemiluminescent HRP Substract, Merck Millipore), and the bands were detected using a photo-documenter.

### Cell apoptosis, proliferation, and viability

Cell apoptosis was estimated with the Fluorescent terminal deoxynucleotidyl transferase nick end labeling of DNA fragments (TUNEL)-in situ cell death detection kit (Fluorescein—Roche), following the manufacturer protocol. Briefly, HUVECs were serum-starved for 4h, followed by stimulation with NCM, HCM, or vehicle control medium for 20h. Only apoptotic cells were stained by TUNEL, while propidium iodide (PI) was used for nuclear labeling. Five hundred cells were counted in randomly chosen fields for each sample, and the numbers of apoptotic cells were expressed as a percentage of the total cells counted.

The relative number of HUVECs incorporating 5-Bromo-2’-deoxyuridine (BrdU), indicating that these cells were going through the S phase, was performed with a cell proliferation kit (Vector). Briefly, HUVECs cultured in EGM-2 medium with 0.5% FBS for 4h were stimulated with NCM, HCM, or vehicle control medium. After 20h, HUVECs were incubated with 10 μM BrdU for 4h, before fixation. BrdU was detected by indirect immunofluorescence staining with a primary mouse anti-Bromodeoxyuridine antibody (1:200, VP-B209, Vector) and secondary anti-IgG mouse conjugate (Alexa Fluor 488, dilution: 1:2000). Images were captured with Eclipse TS100 fluorescence microscopy (Nikon Instruments Inc.). Cell viability was estimated with the fluorescent viability staining calcein-acetoxymethyl ester (calcein-AM; 1 μM; Molecular Probes, Life technologies).

### Cell scratch wound healing assay

The scratch wounds were created in the confluent HUVEC monolayer using a sterile pipette tip, followed by treatment with NCM, HCM, or vehicle control medium for 18h. The wound width was determined at 0h and 18h after scratching using a light microscope equipped with a digital camera. Reference points were marked close to the scratches to evaluate the same field during image acquisition. The open wound area was quantified using the ImageJ (NIH, USA) software. The extent of wound closure was presented as the percentage by which the original scratch width had decreased at each measured time point.

### In vitro 3D sprouting assay

The sprouting assay was carried out as described [33]. Briefly, HUVEC-coated microspheres were resuspended in fibrinogen solution (2.5 mg/mL fibrinogen, Sigma-Aldrich) in EGM-2 medium (without FBS), supplemented with 50 mg/mL aprotinin (Sigma-Aldrich) and plated with 0.15 U thrombin (Sigma-Aldrich) on top of a precoated fibrin layer at 37°C for 20 min. After the gels were allowed to polymerize, NCM, HCM, or vehicle control medium were added and replaced every two days. After four days, the number of endothelial sprouts/beads, branches, and tubule length was quantified in at least 30 microspheres per condition.

### In vivo Matrigel plug assay

In vivo angiogenesis experiments were performed as described [34]. Briefly, a mixture of basement membrane matrix (ice-cold, phenol red-free, reduced growth factor, Gibco) and 10x conditioned medium (0.5 mL, 9:1 proportion) was subcutaneously injected into 8-week-old C57Bl/6 wild-type mice (n = 8, 4 per group). Each mouse received two implants, totaling 8 plugs per group. Buffered saline was included as a negative control during the assay. After 11 days, the plugs were excised, photographed, and processed to assess the angiogenic response. The relative hemoglobin content indicating the degree of blood vessel invasion into the plug was measured as described [31].

### *In vivo* wound healing assay and immunofluorescent labeling

Two cohorts of mice were used in 2 independent experiments (n = 12, 6 per group). Nestin-GFP+/NG2-DsRed+ double-transgenic mice (on the C57BL/6 genetic background) were anesthetized with a combination of ketamine plus xylazine (80 and 20 mg kg^−1^ i.p.) and shaved. Four full-thickness (including the panniculus carnosus) were performed on the back of the mouse, two on each side of the animal midline, by using a 4.0 mm biopsy punch. Wounding day was coded as day 0. Immediately after excision, the wounds on the right side were treated with vehicle control medium and those on the left side were treated with the conditioned medium. One group was treated with normoxic hASC conditioned medium (NCM) and the other group with hypoxic hASC conditioned medium (HCM). A total of 50 μL of conditioned medium (2.5 ± 0.15 mg/mL) or vehicle control medium were subcutaneously injected into four diametrically opposed points at the wound site (1 mm from the wound edges) on days 0 and 2. Wounds were then left uncovered. Digital pictures of wounds were taken at indicated days after wounding. After seven days, mice were euthanized, and the skin sample was harvested, processed, embedded in OCT medium, and stored at—80°C for further immunofluorescence staining, as described [30]. Quantification of blood vessels and perivascular cells was achieved using immunofluorescent visualization of blood vessels on frozen sections. Frozen skin sections were stained with goat anti-CD31 antibody (R&D System, 1:100 in BSA/standard serum solution), rabbit anti-RFP (Abcam, 1:100), chicken anti-GFP (Abcam, 1:100), followed by incubation with the following secondary antibodies: Donkey anti-rabbit 555 (Molecular Probes, 1:500 in BSA/ standard serum solution), Donkey anti-goat 647 (Molecular Probes, 1:500) and Donkey anti-chicken 488 (Jackson Immuno Research, 1:500). Images were captured using fluorescence microscopy analysis (Leika SP8). Quantitative analysis was carried out with the ImageJ (IJ2.3.0/1.53f; Bethesda, MD) software. Colocalizations were assessed with the Leica Application Suite Advanced Fluorescence software. Colocalization was quantified with the RG2B colocalization, Colocalization_Finder, and JACoP plugins of the ImageJ program [35, 36].

### Antibody-based protein array analysis

Analysis of different trophic factors in CM was performed using a Proteome Profiler Human Angiogenesis Array kit (R&D Systems) according to the manufacturer’s instructions. All analyses were performed starting from the same amount of proteins (3mg) for each preparation. Briefly, either 1 mL of NCM or HCM were incubated with the membrane arrays, positive spots were identified by chemiluminescence, and data quantification was performed by densitometry using the ImageJ (NIH, USA) software.

### Functional enrichment of genes associated with regulation of angiogenesis

We used the STRING database (http://www.string-db.org/) to assess the protein-protein interactions (PPIs) of the most abundant proteins identified in our array analysis. The functional enrichment of these PPI by Gene Ontology (GO), including biological process, cellular component, and molecular function, was assessed and visualized with the Cytoscape 3.9.1 software [37]. The hypergeometric test computed the p-values, the minimum required interaction score was 0.7 for high confidence, and the Benjamini & Hochberg false discovery rate (FDR) correction was also defined at a significance level of 0.05.

### Statistical analysis

Statistical analyses were performed with Statistical Package for the Social Sciences (SPSS) version 25.0 software (IBM, Armonk, New York, USA) and GraphPad Prism v6.0 (Graphpad Software, San Diego, California, USA). The significance level (p values) was below 0.05. Quantitative variables distribution was carried out using the Shapiro-Wilk test. Unpaired t-test or Mann–Whitney U tests were used to compare two groups according to the variable distribution, or one-way analysis of variance (ANOVA), followed by the Bonferroni *post-ho*c test for three-group comparisons.

## Results

### Adult hASCs displayed multilineage potential and maintained viability under serum-free medium and hypoxic preconditioning

**Fig 1:**
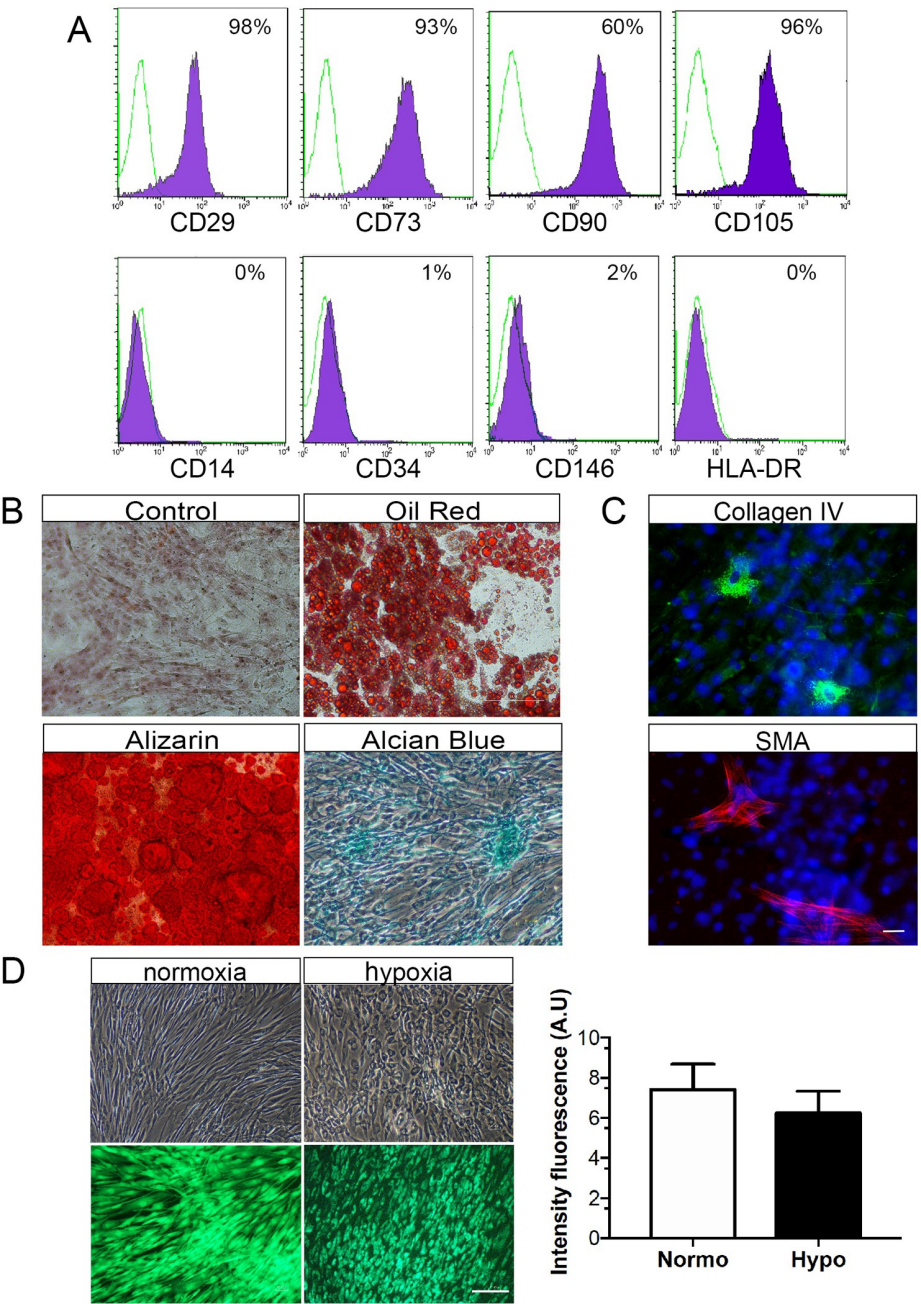
Characterization of hASCs. **(A)** Flow cytometry histograms shows that hASC were positive for CD29, CD73, CD90 and CD105, while negative for CD14, CD34, CD146 and HDLA-DR. The percentage of cells staining for each marker (purple area) and the respective isotype-control (green line) are provided. **(B)** Phase-contrast microscopy imaging of hASCs exposed to adipogenic, osteogenic, or chondrogenic differentiation media. Accumulation of intracellular lipid vacuoles shown by oil red-O staining, calcium-rich extracellular matrix as evidenced by Alizarin red S, and glycosaminoglycans stained with Alcian Blue. **(C)** Immunocytochemistry detection shows α-SMA and collagen-IV-positive hASCs. Nuclei were stained with Hoechst dye (blue). **(D)** After normoxic and hypoxic preconditioning, hASC displayed spindle-shaped fibroblast-like morphology. Staining with Calcein-AM indicated that hASC viability remained unaffected by hypoxic preconditioning. Scale bars: 25μm in C and 75μm in D. Values are expressed as means ± SD of at least three independent experiments (n = 8).

First, adult hASCs were characterized according to their ability to adhere to plastic, the surface antigen markers expression and the multilineage differentiation potential. In culture, the hASCs were constitutively positive for typical mesenchymal markers, with high (>95%) expression of CD29, CD73, CD90 and CD105, and low (< 2%) expression of hematopoietic markers (Fig 1A). The isolated hASC were *in vitro* differentiated in adipogenic, osteogenic and chondrogenic lineages, as showed in Fig 1B. The hASCs expressed low but detectable amounts of α-SMA and collagen-IV as shown in Fig 1C. These data indicated that the isolated hASC consistently meet all basic criteria required for human mesenchymal stem cells derived from lipoaspirate samples, and can be used for subsequent studies. Characterized hASCs cultured in serum-free medium under standard conditions (normoxic) or combined with low oxygen level (hypoxic) preconditioning displayed elongated spindle shapes. Upon hypoxic preconditioning, the viability of hASCs was maintained compared to normoxic condition, as indicated by calcein cell-permeant staining (Fig 1D). These results suggest that 48h serum-free media combined with hypoxic preconditioning did not affect hASCs viability.

### The secretome profile of hASCs is enriched with trophic factors

**Fig 2:**
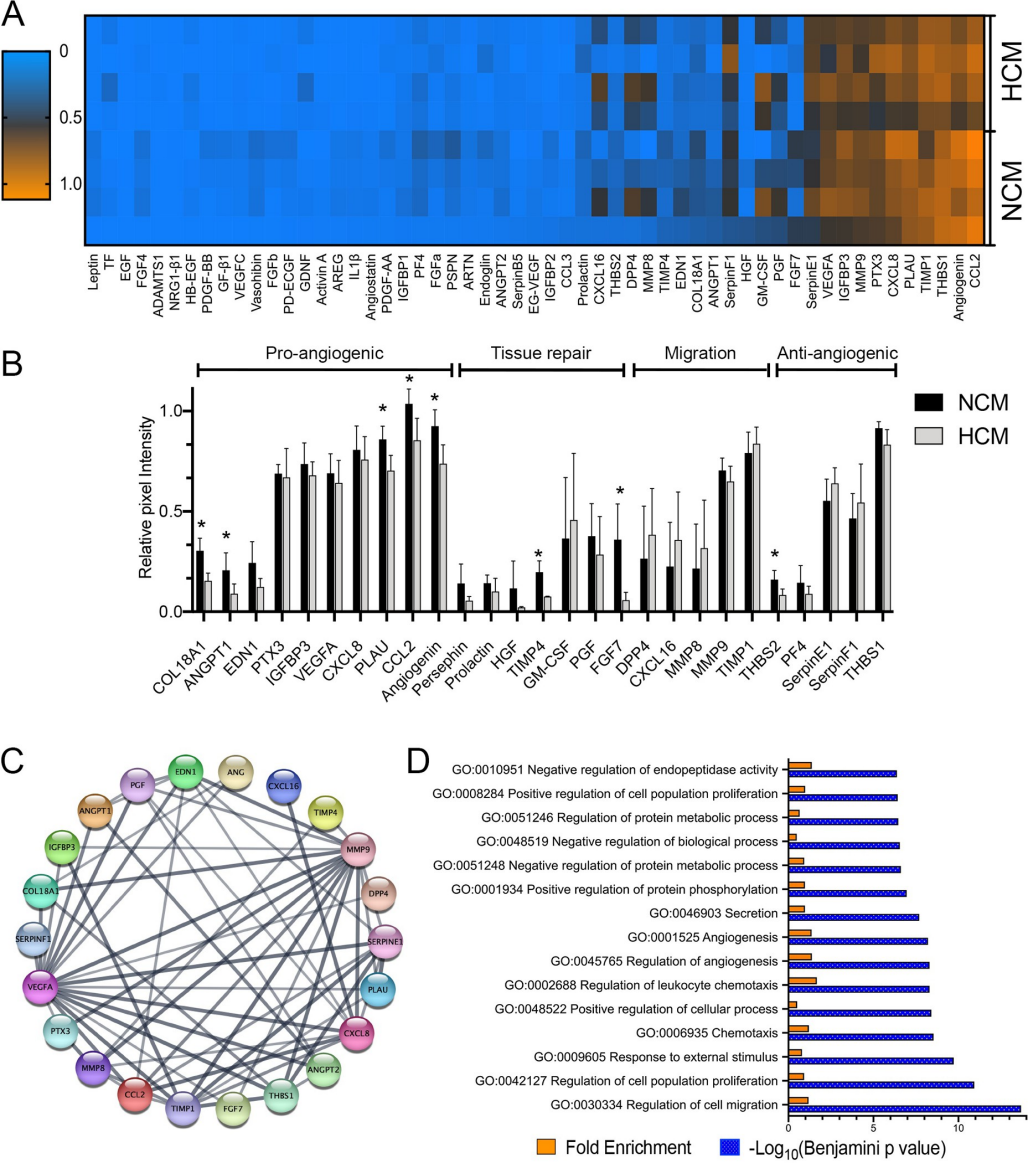
Characterization of HCM and NCM secretomes of human adipose mesenchymal stem cells (hASCs). **(A)** Heatmap depicts the overall profile of 55 trophic factors, cytokines, and chemokines relative to protein abundance (mean pixel intensity). Each column represents a different protein, while each row represents a sample. **(B)** NCM and HCM were analyzed by antibody-based protein array kit. Values are normalized to positive reference spots. Data are representative of three independent experiments, and values are expressed in mean ± SD. **(C)** STRING analysis uncovering protein-protein interaction (PPI) network of most abundant trophic factors expressed in NCM and HCM visualized by Cytoscape. **(D)** David Gene Ontology analyses of proteins more abundant in hASC secretome. GO enrichment analysis showed the biological process of most representative trophic factors ranked by p-value. The top 15 processes were selected based on the Benjamini p-value (− Log_10_ Benjamini p-value are reported as blue bars). Fold enrichment is also reported as orange bars. * p<0.05. N = 8.

Analysis of the conditioned medium revealed that of 55 angiogenic-related mediators analyzed, 27 were consistently detected on the membrane in at least three samples of at least one group (Fig 2A). A total of 12 growth factors/cytokines with relevant expression (OD Abs > 0.5) were expressed in both CMs, and eight trophic factors were differentially expressed between NCM and HCM (Fig 2B). Since this result indicated the presence of both pro-and antiangiogenic factors, we identified enriched pathways and biological processes in the whole secretome. The list of consistently more abundant proteins in NCM and HCM samples was analyzed to predict a protein-protein interaction network (PPI) with the String-online database (Fig 2C). GO enrichment analysis showed that the more abundant proteins played an essential role in angiogenesis, vasculature development, cell migration, and tissue repair (Figs 2D and S1A). The KEGG pathway enrichment analysis results showed that the differentially expressed proteins were mainly involved in the pathways related to response to hypoxia, cell proliferation, and migration, including PI3K-Akt, HIF-1, Rap-1 signaling cytokine-cytokine receptor interaction pathways (S1B Fig).

### hASCs-conditioned medium maintained viability and induced proliferation of HUVEC

**Fig 3:**
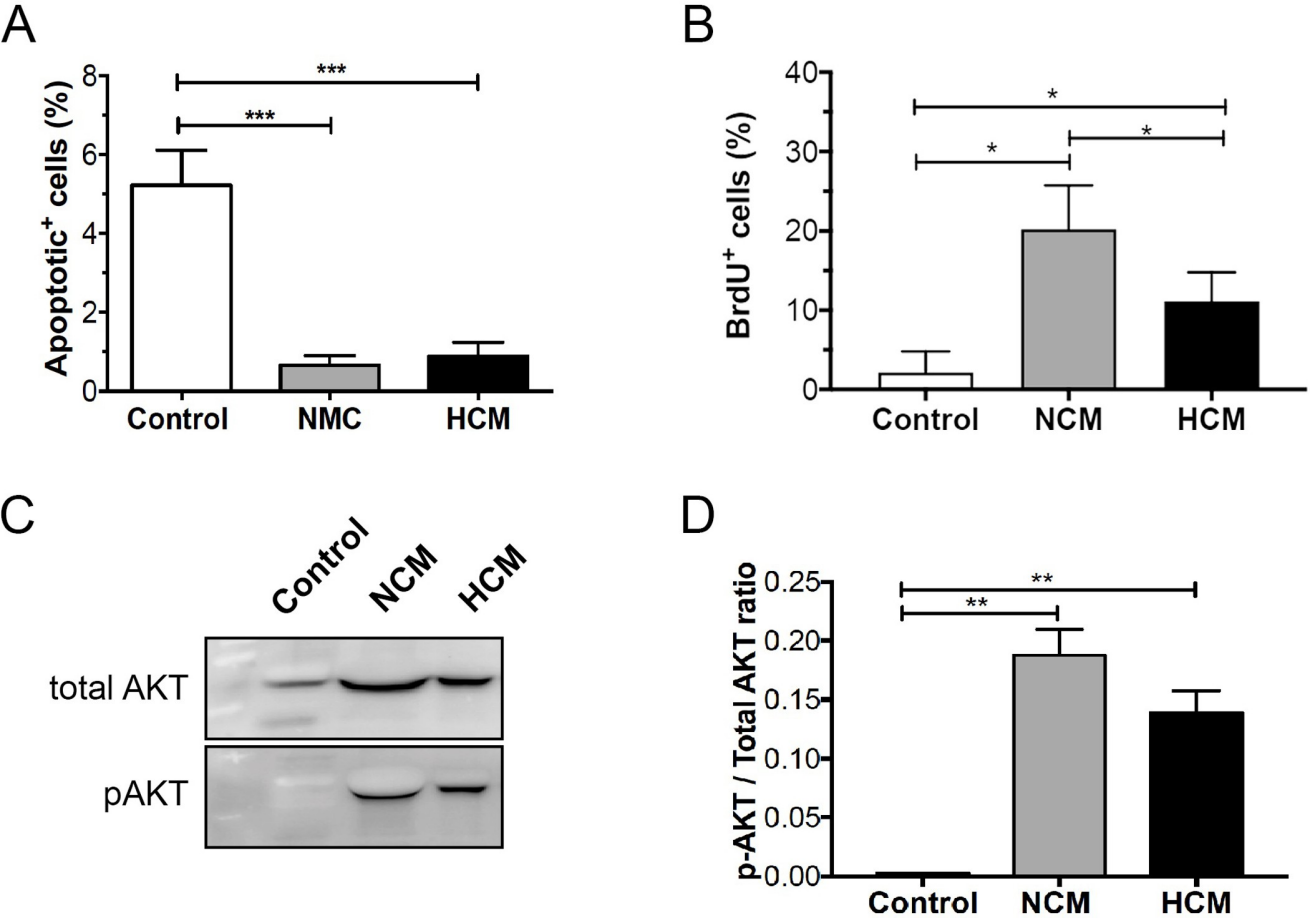
Normoxic- and hypoxic- hASCs secretomes increase cell proliferation and attenuate apoptosis in HUVECs. **(A)** HUVECs were cultured for 24h in medium alone (control) or supplemented with either NCM or HCM. TUNEL-positive cells were counted, and the apoptotic index was calculated as the average number of positive cells compared to the total number of cells in at least six visual fields. Values shown are the mean ± SD of at least three independent experiments. **(B)** HUVECs were pulse-labeled with 10 μM BrdU, cultured for up to 24 hours in the presence of NCM or HCM, and BrdU was visualized by immunocytochemistry and quantified by cell counting. **(C)** Representative western blots of pAKT and total AKT expression in HUVECs exposed to NCM or HCM. **(D)** Quantitative data representing the average values of three independent experiments. The Fig 3C was cropped to improve the clarity and conciseness of the presentation, and the full-length blots are presented in S2 Fig. Results indicate mean normalized expression relative to control ± SD. Cell experiment was repeated three times independently. *p<0.05; ** p<0.01 (n = 4).

To verify the *in vitro* effects of NCM and HCM on the viability of endothelial cells, we performed the TUNEL assay. The experiment indicated a decreased number of late apoptotic cells in HUVECs treated with NCM or HCM when compared to the control group (0.6 ± 0.7, 0.8 ± 1.0 vs 5.2 ± 2.6, p<0.05), indicating that both conditioned media exhibited antiapoptotic characteristics and had a positive effect on cell survival (Fig 3A). Next, we evaluated the incorporation of BrdU in HUVECs treated with NCM or HCM. After 24h, a significant number of BrdU-positive HUVECs was observed in the presence of NCM and HCM when compared with the control group (p<0.05). These results indicate that NCM and HCM were able to induce proliferation in HUVECs (Fig 3B). Various signaling pathways, including PI3K-AKT, have been implicated in the pro-angiogenic function of endothelial cells. Accordingly, we tested if NCM and HCM conditioned media induced intracellular signaling in HUVECs. Serum and growth factor starved HUVECs were stimulated in a time-dependent manner. We found that serum-starved HUVECs stimulated with NCM or HCM activated the PI3K-AKT pathway. Both conditioned media induced activation of the AKT pathway after 5min (p<0.05) (Figs 3C and 3D and S1). These data showed that both CMs were able to activate the PI3K-AKT pathway, which is involved in cell survival mechanisms, corroborating the predicted PPI network and previous results about the effect of NCM and HCM on endothelial cell survival and proliferation.

### hASCs-conditioned medium stimulates migration and sprouting of endothelial cells

**Fig 4:**
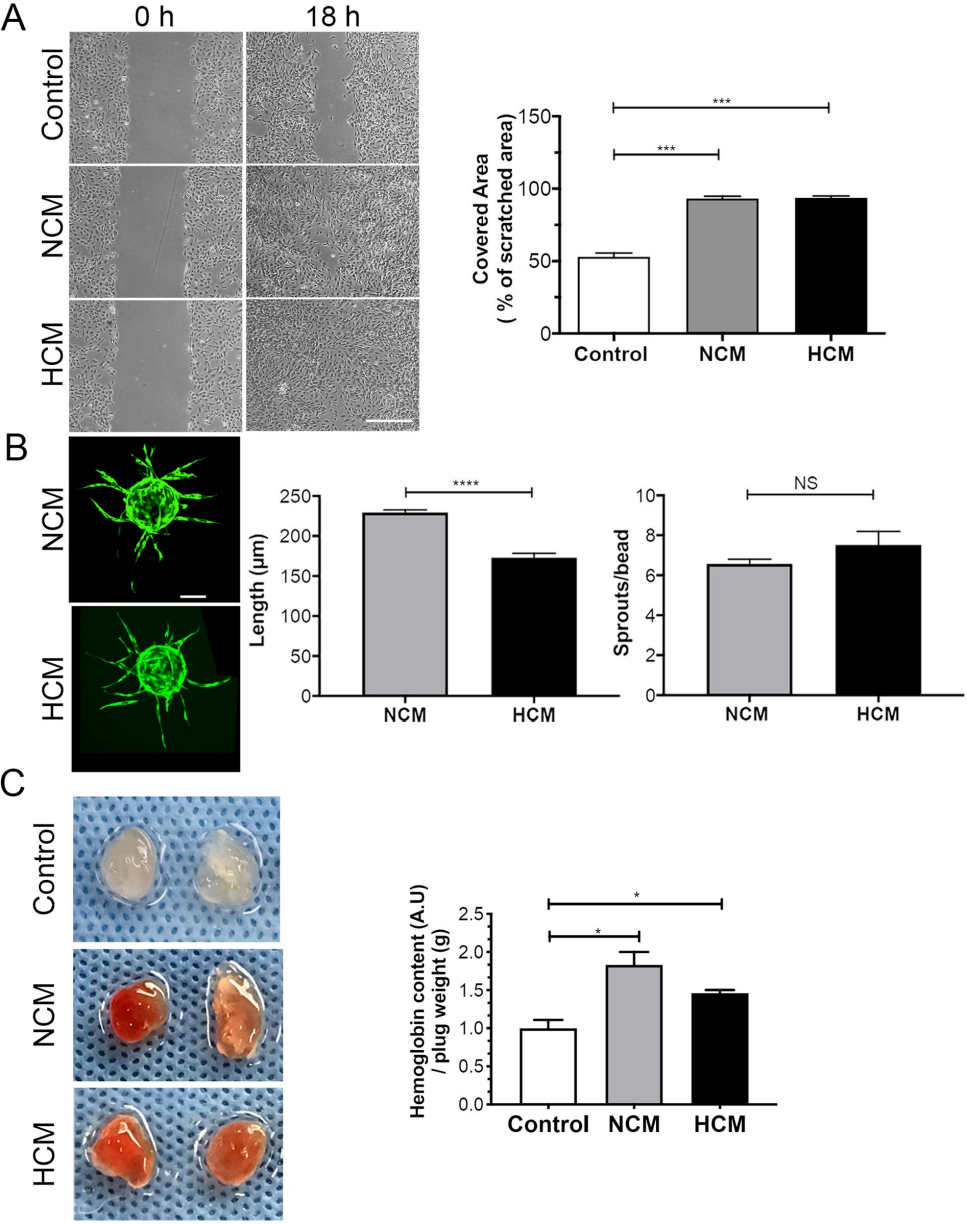
Hypoxic- (HCM) and normoxic- (NCM) hASCs secretomes accelerate *in vitro* cell migration and show *in vitro* and *in vivo* pro-angiogenic potential. **(A)** Representative image of the migration of HUVEC cells after 0 or 18 h of incubation in HCM, NCM or control. Quantification of the percentage of variation in the wound area (% migration area). Scale bars = 250 μm **(B)** Representative images of EC-coated beads with NCM or HCM on fibrin gel and stained with calcein. Quantitation of EC sprouting formation and sprout length in arbitrary units formed after 4 days. Scale bars = 100μm **(C)** Mixture of matrigel-filled plugs containing indicated conditioned medium (NCM or HCM) or PBS (control) were injected subcutaneously in mice (n = 8). After 11 days, hemoglobin content representative of invading vessels was measured. The two-way ANOVA test and the Bonferroni post-test were used to analyze the differences among the groups. Values are expressed as mean ± SD of at least three independent experiments. (C) **p<0.01; ***p<0.001 (n = 12, 6 per group).

Angiogenesis is a multistep process that requires cell migration, proliferation, survival, and tube formation. We evaluated whether the hASCs-conditioned medium stimulates angiogenesis. First, the chemotactic potential of the conditioned medium was examined with the *in vitro* scratch wound assay. HUVEC migration was significantly enhanced (~2.0-fold) in the presence of NCM and HCM, in comparison to the vehicle control medium (99.5 ± 0.5; 99.2 ± 0.9 vs 55.5 ± 8.5% of original scratched area, *** p<0.0005), with monolayer scratching 100% recovered after 18h (Fig 4A). Next, *in vitro* angiogenesis was examined in HUVECs seeded on collagen-coated Cytodex beads embedded in a fibrin gel. Under control conditions, no sprouts were formed, and this group was removed from the analyzed graphs. In the presence of either NCM or HCM, endothelial sprouts appeared around day 2, and capillary-like structures were formed around day 6. Fig 4B exhibits the formation of a more significant number of endothelial sprouts/beads (6.5 ± 1.6 vs 5.0 ± 2.8 sprouts/beads, p<0.05) with greater lengths (229.4 ± 49.6 vs 172.8 ± 73.7 μm, p<0.0001) in the presence of NCM, as compared to HCM. A similar response was observed in the Matrigel plug assay to evaluate *in vivo* angiogenesis. Both NCM or HCM conditioned media were able to induce invasion of blood vessels into the Matrigel plug when compared to control (p<0.05) (Fig 4C). These results indicate that NCM and HCM showed pro-angiogenic capacity, and hypoxia preconditioning did not alter the effects of hASCs.

### The hASC-derived conditioned medium enhances skin repair in a wound healing mouse model

**Fig 5:**
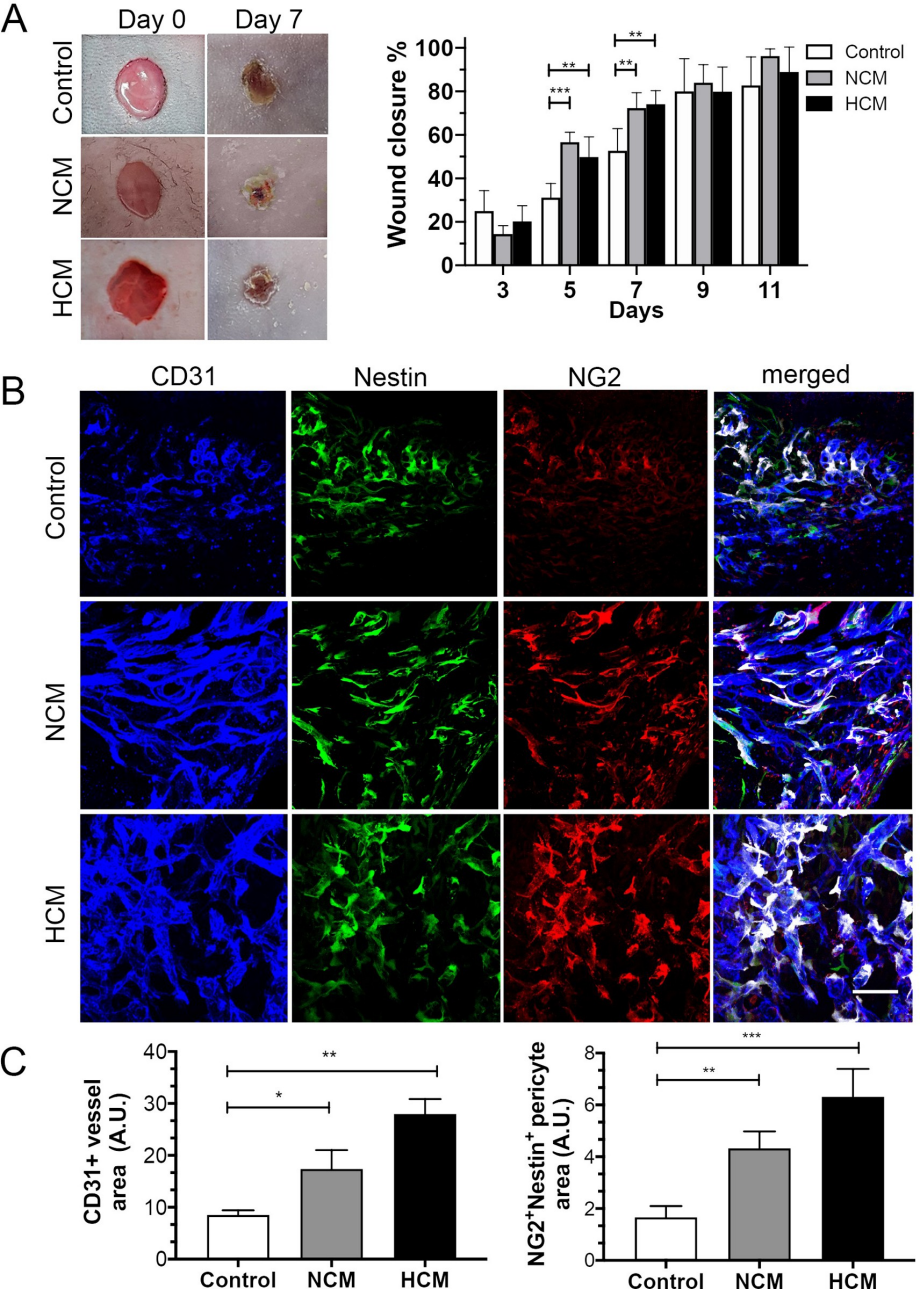
NG2+Nestin+ pericytes surrounding the blood vessels and in close contact with the vascular wall in hASCs secretomes treated animals. **(A)** Representative macroscopic images showing cutaneous wounds on days 0 and 7 after injection of control, hypoxic- (HCM) or normoxic- (NCM) secretomes. **(B)** HCM and NCM accelerated wound closure and microvessel density. Confocal images of the skin wound sections labeled NG2+(red)/Nestin+(green) pericytes and CD31+ blood vessels (blue). Use of pseudocolor (white) to display colocalization of NG2+(red) and nestin+(green) pericytes around CD31+microvessels (blue). **(C)** The extent of microvessel density was determined by assessing the CD31+ vessel area or NG2+nestin+ area in each of 4 randomly chosen high-power fields within the injury site. Scale bar, 100 μm for images in (B). Results are given as the means ± the SD. *p<0.05, **p<0.01; ***p<0.001 (n = 12, 6 per group).

The reparative potential of the conditioned medium was evaluated in a murine full-thickness excisional wound healing model. The wounds were treated with a control medium, NCM, or HCM, and observed on days 0, 3, 5, 7, 9, and 11. On day 7, the wound healing process was accelerated in wounds exposed to NCM and HCM, when compared with the vehicle control group, with a reduction of the injured area on days 9 and 11 (p<0.05) (Fig 5A). The relative abundance of CD31+ (endothelial cell marker) and Nestin+NG2+ perivascular (pericyte cell markers) populations were analyzed in wounded skin (Fig 5B). Upon injury, our immunofluorescence staining demonstrated that NCM or HCM treatment led to a significant increase in CD31+ capillaries. These CD31+ capillaries exhibited a higher percentage of endothelial vessels associated with pericytes when compared to the control medium (p<0.05) (Fig 5C). These results suggest that human hASCs secretome enhances endothelial blood vessel formation and pericyte coverage during full-thickness excisional skin repair.

## Discussion

Emerging evidence shows that the therapeutic effects of hASCs result from their intense paracrine activity mediated by their secretome. However, hASCs represent a heterogeneous cell population with varied secretory behavior. hASCs preconditioning is a valuable alternative to improve secretome function and reduce inter-individual cell variability [38, 39]. The present study used serum-free medium alone or combined with hypoxia as preconditioning strategies to yield normoxic (NCM) or hypoxic (HCM) secretomes, respectively. We showed that both secretomes enhanced endothelial cell proliferation and migration and reduced endothelial cell apoptosis. Additionally, in vitro endothelial sprouting and *in vivo* Matrigel invasion were almost equivalent for both normoxic and hypoxic secretomes. Moreover, we demonstrated that both normoxic and hypoxic hASCs secretomes accelerated wound healing, blood vessel formation, and resident pericyte coverage in the murine full-thickness excisional wound healing model. These findings suggest that serum-free media or hypoxia preconditioning promoted the angiogenic and skin repair potential of hASCs secretomes.

Several studies indicate that serum-free media may benefit mesenchymal stem cells proliferation, differentiation, and paracrine activity while maintaining their stemness, immunosuppressive and antifibrotic abilities [40–42]. In general, secretomes collected under serum-free conditions are much more appropriate for clinical use because of reduced contamination and safety concerns related to xenogeneic infectious agents [43–45]. Serum-free conditions facilitate the proteomic analysis of secretomes [5, 46]. In addition, hypoxic preconditioning has been widely studied to improve hASCs paracrine activity in models in which proangiogenic and skin-repairing effects of secretomes are expected [4, 47]. Therefore, we investigate whether serum-free media alone or combined with hypoxic preconditioning constituted an effective strategy to increase the number and function of secreted molecules. The present study found that serum-free condition induces paracrine activity in hASCs to produce a relatively enriched secretome. In addition, the combination of serum-free condition and hypoxia preconditioning has a limited effect on the secretome composition. Our results further indicated that serum-free media and hypoxia preconditioning for 48h did not affect hASC’s spindle-shaped cell morphology and viability, consistent with previous studies [40, 47, 48]. The predicted PPI and the protein array profiling of NCM and HCM secretomes showed that the synergism employed here caused no substantial alteration of the paracrine factor landscape, with only minimal significant differences between these two types of secretomes. Similarly, Peltzer et al. [39] showed that hypoxia preconditioning has no additional effect on mesenchymal paracrine activity primed with Interferon-g, and only a limited number of secreted molecules was affected by hypoxia. Moreover, Ferreira et al. [20] described that hypoxia preconditioning for short periods significantly impacted cell proliferation and increased ASCs cell survival and paracrine ability. These discrepancies are likely due to experimental conditions or donor inter-variability [49]. Therefore, our results suggest that hypoxic preconditioning has no additional effect on the serum-free media condition to improve the secretome composition.

hASCs secretomes enriched with angiogenic and other paracrine growth factors display a notable function to accelerate healing and promote wound angiogenesis in various preclinical animal models. In our study, both NCM and HCM secretomes showed strong angiogenic and healing potential, as evidenced by HUVEC proliferation, sprouting formation, and *in vivo* angiogenesis. In this context, we analyzed hASCs secretomes to identify possible paracrine factors that are functionally involved in the acceleration of wound healing processes. Predominantly, we identified regulators of tissue repair, including pro- (VEGF-A, angiogenin, IGFBP3) and anti-angiogenic (Serpins F1 and E1, THBS1) factors, inflammatory response mediators (CCL2, CXCL8, PTX3), migration (MMP-9 and TIMP-1) and coagulation (PLAU) factors. Recently, Cases-Perera et al., [50] identified 8 proteins enriched in hASCs secretome, including VEGF, TIMP-1, THBS1, Serpin F1/E1, IGFBP-3, and PTX3. Remarkably, our results also indicated high expression of CCL2, Angiogenin, CXCL8, that are critical players with proangiogenic effect, as previously described by Kim et al., [11]. Although the composition of hASCs secretomes have differed slightly with culture preconditioning, the presence of these proteins in similar amount suggests that their potential role are equivalents [50]. These results are in agreement with previous reports suggesting that hASCs secretome are an abundant source of trophic factors, with marked growth and angiogenic properties—factors, that could be relevant to accelerate wound healing and enhance in vivo skin repair [3, 11, 50].

Even though available reports state that under standard culture conditions, hASCs secrete high levels of VEGF-A and other proangiogenic factors [51, 52], which can be even higher under low oxygen conditions [53, 54], we found equivalent expression levels of VEGF-A in both normoxic and hypoxic secretomes. The serum-free condition has been shown to induce a stress response that might be sufficient to stimulate a paracrine activity in hASCs, rendering uncertain the effect of the low oxygen tension [55, 56]. Similarly, a recent report has shown that normoxic or hypoxic hASCs secretomes contain equivalent levels of angiogenin, VEGFA, CCL2, and IGFBP3 and accelerate the healing process by increasing fibroblast migration and granulation tissue formation [57]. Additionally, after mass spectrometry analysis of hASCs secretome, Riis and colleagues [24] could not detect a significant effect of hypoxic preconditioning. Only a relatively small fraction (9.6%) of the proteome was affected by low oxygen compared to normoxia and serum-free conditions [24]. From this perspective, An et al. [52] analyzed the proteomic profile of serum-free hASCs secretome. They revealed a remarkably high amount of protein molecules for wound healing, i.e., TGF-β1 and VEGF, besides more than 700 proteins highly involved in the extracellular matrix organization, angiogenesis, and cell migration. In accordance, a comprehensive quantitative proteomics approach recently explored the protein composition of serum-free hASC secretome and identified more than 1977 proteins involved in ECM organization (hyaluronan and glycosaminoglycan metabolism) and immunological regulation (e.g., macrophage and IkB/NFkB signaling regulation) [5].

Among the in-silico hASCs-enriched processes, platelet degranulation, extracellular matrix organization, and regulation of smooth muscle cell-matrix adhesion pathways emerged. Our *in vivo* results demonstrated that normoxic or hypoxic hASCs secretome enhanced blood vessel density and pericyte coverage during wound healing. These results are consistent with other preclinical studies of skin repair, demonstrating that paracrine factors increase vessels density and accelerate healing in full-thickness excisional wounds [58, 59], diabetic wounds [60–62], hypertrophic and keloid scars [63], skin flaps [64] and hair loss [65]. However, it is noteworthy that most studies do not assess the presence of perivascular cells (i.e., pericytes) surrounding the newly formed blood vessels mediated by the hASCs secretomes. Pericytes play a pivotal role in maintaining vascular integrity and restoring skin function after acute injury [66]. In this context, our results completely align with previously described results for wound angiogenesis and skin repair [67].

In our study, we found that nestin+/NG2+ pericytes were surrounding blood vessels seven days post-wound. We also quantified perivascular cells, and found the highest number of nestin+/NG2+ pericytes in wounds treated with hASC secretome and accelerated healing. It is suggestive that these perivascular cells may help vessel stabilization, maturation, or vessel remodeling. These results were consistent with previous observations that nestin+/NG2+ pericytes generate blood vessel tissues [27, 68], and NG2+ perivascular cells are co-localized with blood vessels for up to 10 days post-wound [69]. Recently, do Valle et al. [67] demonstrated that an increased number of nestin+/NG2+ pericytes and undifferentiated cells were mobilized to wound edges in the initial experimental periods and accumulated in the dermal regions of the wound afterward. Further work is needed to explore the role of nestin+/NG2+ pericytes in stabilizing blood vessels, contributing to vascular maturation, remodeling, and regulating the permeability and blood flow in wound healing. The healing effect accomplished by hASCs secretome presumably was because it provided essential trophic and mediating factors that helped create a proangiogenic response at different stages of the wound healing process. However, it remains unclear whether hASCs secretome contributes to the direct activation of pericytes and endothelial cells or is indirectly responsible for the recruitment of these and other cells (tissue-resident progenitors, inflammatory cells) due to metalloproteinases and chemokines detected in secretomes.

hASCs secretomes are currently in phase I and II clinical trials for diverse applications [70–72], even though the road toward an exhaustive characterization of this new generation of cell-free therapeutic is still a very long one. Compared to cell therapy, the use of secretomes has lower biological risks, less tumorigenicity and reduced cost [3]. However, the risk of disease transmission as well as xenogeneic immune reactions in the recipient is a clear limitation. In addition, the commercial use of secretome still requires challenges to be addressed, such as the optimization and reproducibility of production methods, better characterization of its constituents (content of cytokines, growth factors, microRNAs, lipids and microvesicles), better preservation and dose-effect evaluation to achieve the best efficacy profile for clinical applications. The use of filter sterilization to remove biological contaminants during the preparation of commercially intended hASCs secretomes should be carefully evaluated, as it could considerably affect the contents of microvesicles and extravelllar vesicles with therapeutic potential [3, 4].

In conclusion, our results suggest that serum-free media or hypoxic preconditioning hASCs secretomes display a very similar composition of specific bioactive factors. Both NCM and HCM were potent in promoting *in vitro* and *in vivo* angiogenesis, tissue vascularization, and vascular coverage of resident pericytes expressing NG2 and nestin to the lesion site, potentially contributing to blood vessel maturation. Overall, normoxic and hypoxic preconditioning enhanced the vascular reparative effects of hASCs secretome on the preclinical wound healing model. Therefore, from the clinical translation perspective, hASCs secretome represents a potential therapeutic product in a practical and minimal manipulation procedure, affording a more feasible scale-up approach.

## Supporting information

S1 Fig (A) KEGG enriched pathways of most representative trophic factors in hASC secretome ranked by p-value. − Log10 Benjamini p-value are reported as blue bar and Fold enrichment as orange bars. (B) Gene Ontology analyses of proteins more abundant in hASC conditioned medium ranked by Fold enrichment. The top 25 processes are shown.
(TIFF)

S2 Fig Total AKT and phospho-AKT(Ser473) were detected in HUVECs after incubations with hASC secretomes. Representative Western blots for total-Akt (A) and P-Akt_Ser473_ (B) from HUVECs that were starved in 0.1%BSA EBM-2 and stimulated with control medium, NCM or HCM for 5 min.
(JPG)

S1 Raw images phospho-AKT(Ser473). (TIF)

S2 Raw images Total AKT. (TIFF)

S1 Table Patient and tissue harvesting information. (DOCX)

## References

[1] Liu X, Wang Z, Wang R, Zhao F, Shi P, Jiang Y, et al. Direct comparison of the potency of human mesenchymal stem cells derived from amnion tissue, bone marrow and adipose tissue at inducing dermal fibroblast responses to cutaneous wounds. Int J Mol Med. 2013;31(2):407–15. doi: 10.3892/ijmm.2012.1199 . 23228965

[2] Riedl J, Popp C, Eide C, Ebens C, & Tolar J. Mesenchymal stromal cells in wound healing applications: role of the secretome, targeted delivery and impact on recessive dystrophic epidermolysis bullosa treatment. Cytotherapy. 2021;23(11):961–973. doi: 10.1016/j.jcyt.2021.06.004 . 34376336 PMC8569889

[3] Ahangar P, Mills SJ, & Cowin AJ. Mesenchymal Stem Cell Secretome as an Emerging Cell-Free Alternative for Improving Wound Repair. Int J Mol Sci. 2020;21(19):7038. doi: 10.3390/ijms21197038 . 32987830 PMC7583030

[4] Ajit A, & Ambika Gopalankutty I. Adipose-derived stem cell secretome as a cell-free product for cutaneous wound healing. 3 Biotech. 2021;11(9):413. doi: 10.1007/s13205-021-02958-7 . 34476171 PMC8368523

[5] Niada S, Giannasi C, Magagnotti C, Andolfo A, & Brini AT. Proteomic analysis of extracellular vesicles and conditioned medium from human adipose-derived stem/stromal cells and dermal fibroblasts. J Proteomics. 2021;232:104069. doi: 10.1016/j.jprot.2020.104069 . 33309826

[6] Montero-Vilchez T, Sierra-Sánchez Á, Sanchez-Diaz M, Quiñones-Vico MI, Sanabria-de-la-Torre R, Martinez-Lopez A, et al. Mesenchymal Stromal Cell-Conditioned Medium for Skin Diseases: A Systematic Review. Front Cell Dev Biol. 2021;9:654210. doi: 10.3389/fcell.2021.654210 . 34368115 PMC8343397

[7] Ratajczak MZ, Kucia M, Jadczyk T, Greco NJ, Wojakowski W, Tendera M, et al. Pivotal role of paracrine effects in stem cell therapies in regenerative medicine: can we translate stem cell-secreted paracrine factors and microvesicles into better therapeutic strategies? Leukemia. 2012;26(6):1166–73. doi: 10.1038/leu.2011.389 . 22182853

[8] Bellei B, Migliano E, Tedesco M, Caputo S, Papaccio F, Lopez G, et al. Adipose tissue-derived extracellular fraction characterization: biological and clinical considerations in regenerative medicine. Stem. Cell. Res. Ther. 2018;9(1):207. doi: 10.1186/s13287-018-0956-4 . 30092820 PMC6085647

[9] Trzyna A, & Banaś-Ząbczyk A. Adipose-Derived Stem Cells Secretome and Its Potential Application in "Stem Cell-Free Therapy". Biomolecules. 2021;11(6):878. doi: 10.3390/biom11060878 . 34199330 PMC8231996

[10] Park SR, Kim JW, Jun HS, Roh JY, Lee HY, & Hong IS. Stem Cell Secretome and Its Effect on Cellular Mechanisms Relevant to Wound Healing. Mol. Ther. 2018;26(2):606–617. doi: 10.1016/j.ymthe.2017.09.023 . 29066165 PMC5835016

[11] Kim HK, Lee SG, Lee SW, Oh BJ, Kim JH, Kim JA, et al. A Subset of Paracrine Factors as Efficient Biomarkers for Predicting Vascular Regenerative Efficacy of Mesenchymal Stromal/Stem Cells. Stem Cells. 2019;37(1):77–88. doi: 10.1002/stem.2920 . 30281870

[12] Liang X, Zhang L, Wang S, Han Q, & Zhao RC. Exosomes secreted by mesenchymal stem cells promote endothelial cell angiogenesis by transferring miR-125a. J. Cel.l Sci. 2016;129(11):2182–9. doi: 10.1242/jcs.170373 . 27252357

[13] Gong M, Yu B, Wang J, Wang Y, Liu M, Paul C, et al. Mesenchymal stem cells release exosomes that transfer miRNAs to endothelial cells and promote angiogenesis. Oncotarget. 2017;8(28):45200–45212. doi: 10.18632/oncotarget.16778 . 28423355 PMC5542178

[14] Waters R, Subham S, Pacelli S, Modaresi S, Chakravarti AR & Paul A. Development of MicroRNA-146a-Enriched Stem Cell Secretome for Wound-Healing Applications. Mol. Pharm. 2019;16(10):4302–4312. doi: 10.1021/acs.molpharmaceut.9b00639 . 31398053 PMC7260687

[15] Ghafouri-Fard S, Niazi V, Hussen BM, Omrani MD, Taheri M, & Basiri A. The Emerging Role of Exosomes in the Treatment of Human Disorders with a Special Focus on Mesenchymal Stem Cells-Derived Exosomes. Front. Cell. Dev. Biol. 2021; 9:653296. doi: 10.3389/fcell.2021.653296 . 34307345 PMC8293617

[16] Noronha NC, Mizukami A, Caliári-Oliveira C, Cominal JG, Rocha JLM, Covas DT, et al. Priming approaches to improve the efficacy of mesenchymal stromal cell-based therapies. Stem Cell. Res. Ther. 2019;10(1):131. doi: 10.1186/s13287-019-1224-y . 31046833 PMC6498654

[17] Bachmann J, Ehlert E, Becker M, Otto C, Radeloff K, Blunk T, et al. Ischemia-Like Stress Conditions Stimulate Trophic Activities of Adipose-Derived Stromal/Stem Cells. Cells. 2020;9(9):1935. doi: 10.3390/cells9091935 . 32825678 PMC7566001

[18] Sun B, Guo S, Xu F, Wang B, Liu X, Zhang Y, et al. Concentrated Hypoxia-Preconditioned Adipose Mesenchymal Stem Cell-Conditioned Medium Improves Wounds Healing in Full-Thickness Skin Defect Model. Int. Sch. Res. Notices. 2014; 2014:652713. doi: 10.1155/2014/652713 . 27433483 PMC4897251

[19] Choi JR, Yong KW, & Wan Safwani W. Effect of hypoxia on human adipose-derived mesenchymal stem cells and its potential clinical applications. Cell. Mol. Life Sci. 2017;74(14):2587–2600. doi: 10.1007/s00018-017-2484-2 . 28224204 PMC11107561

[20] Ferreira JR, Teixeira GQ, Santos SG, Barbosa MA, Almeida-Porada G, & Gonçalves RM. Mesenchymal Stromal Cell Secretome: Influencing Therapeutic Potential by Cellular Pre-conditioning. Front. Immunol. 2018; 9:2837. doi: 10.3389/fimmu.2018.02837 . 30564236 PMC6288292

[21] Almeria C, Weiss R, Roy M, Tripisciano C, Kasper C, Weber V, et al. Hypoxia Conditioned Mesenchymal Stem Cell-Derived Extracellular Vesicles Induce Increased Vascular Tube Formation in vitro. Front. Bioeng. Biotechnol. 2019; 7:292. doi: 10.3389/fbioe.2019.00292 . 31709251 PMC6819375

[22] Li Y, Zhang W, Gao J, Liu J, Wang H, Li J, et al. Adipose tissue-derived stem cells suppress hypertrophic scar fibrosis via the p38/MAPK signaling pathway. Stem Cell. Res. Ther. 2016;7(1):102. doi: 10.1186/s13287-016-0356-6 . 27484727 PMC4970202

[23] Li M, Luan F, Zhao Y, Hao H, Liu J, Dong L, et al. Mesenchymal stem cell-conditioned medium accelerates wound healing with fewer scars. Int. Wound J. 2017;14(1):64–73. doi: 10.1111/iwj.12551 . 26635066 PMC7949734

[24] Riis S, Stensballe A, Emmersen J, Pennisi CP, Birkelund S, Zachar V, et al. Mass spectrometry analysis of adipose-derived stem cells reveals a significant effect of hypoxia on pathways regulating extracellular matrix. Stem Cell. Res. Ther. 2016;7(1):52. doi: 10.1186/s13287-016-0310-7 . 27075204 PMC4831147

[25] Lee MS, Youn C, Kim JH, Park BJ, Ahn J, Hong S, et al. Enhanced Cell Growth of Adipocyte-Derived Mesenchymal Stem Cells Using Chemically-Defined Serum-Free Media. Int. J. Mol. Sci. 2017;18(8):1779. doi: 10.3390/ijms18081779 . 28813021 PMC5578168

[26] Ma H, Lam PK, Siu WS, Tong CSW, Lo KKY, Koon CM, et al. Adipose Tissue-Derived Mesenchymal Stem Cells (ADMSCs) and ADMSC-Derived Secretome Expedited Wound Healing in a Rodent Model—A Preliminary Study. Clin. Cosmet. Investig. Dermatol. 2021;14:753–764. doi: 10.2147/CCID.S298105 . 34234501 PMC8255652

[27] Birbrair A, Zhang T, Wang ZM, Messi ML, Olson JD, Mintz A, et al. Type-2 pericytes participate in normal and tumoral angiogenesis. Am. J. Physiol. Cell. Physiol. 2014;307(1):C25–38. doi: 10.1152/ajpcell.00084.2014 . 24788248 PMC4080181

[28] Silva KR, Liechocki S, Carneiro JR, Claudio-da-Silva C, Maya-Monteiro CM, Borojevic R, et al. Stromal-vascular fraction content and adipose stem cell behavior are altered in morbid obese and post bariatric surgery ex-obese women. Stem Cell. Res. Ther. 2015;6(1):72. doi: 10.1186/s13287-015-0029-x . 25884374 PMC4435525

[29] Crampton SP, Davis J & Hughes CC. Isolation of human umbilical vein endothelial cells (HUVEC). J. Vis. 2007;(3):183. doi: 10.3791/183 . 18978951 PMC2576276

[30] Ribeiro TO, Silveira BM, Meira MC, Carreira ACO, Sogayar MC, Meyer R, et al. Investigating the potential of the secretome of mesenchymal stem cells derived from sickle cell disease patients. PLoS One. 2019;14(10):e0222093. doi: 10.1371/journal.pone.0222093 . 31665139 PMC6821040

[31] Santos GC, Silva DN, Fortuna V, Silveira BM, Orge ID, de Santana TA, et al. Leukemia Inhibitory Factor (LIF) Overexpression Increases the Angiogenic Potential of Bone Marrow Mesenchymal Stem/Stromal Cells. Front. Cell. Dev. Biol. 2020;8:778. doi: 10.3389/fcell.2020.00778 . 32923442 PMC7456813

[32] Overath JM, Gauer S, Obermüller N, Schubert R, Schäfer R, Geiger H, et al. Short-term preconditioning enhances the therapeutic potential of adipose-derived stromal/stem cell-conditioned medium in cisplatin-induced acute kidney injury. Exp. Cell. Res. 2016;342(2):175–83. doi: 10.1016/j.yexcr.2016.03.002 . 26992633

[33] Nakatsu MN & Hughes CC. An optimized three-dimensional in vitro model for the analysis of angiogenesis. Methods Enzymol. 2008; 443:65–82. doi: 10.1016/S0076-6879(08)02004-1 . 18772011

[34] Malinda KM. In vivo matrigel migration and angiogenesis assay. Methods Mol. Biol. 2009; 467:287–94. doi: 10.1007/978-1-59745-241-0_17 19301678

[35] Bolte S & Cordelières FP. A guided tour into subcellular colocalization analysis in light microscopy. J. Microsc. 2006;224(Pt 3):213–32. doi: 10.1111/j.1365-2818.2006.01706.x . 17210054

[36] Dunn KW, Kamocka MM & McDonald JH. A practical guide to evaluating colocalization in biological microscopy. Am. J. Physiol. Cell. Physiol. 2011;300(4):C723–42. doi: 10.1152/ajpcell.00462.2010 . 21209361 PMC3074624

[37] Doncheva NT, Morris JH, Gorodkin J & Jensen LJ. Cytoscape StringApp: Network Analysis and Visualization of Proteomics Data. J. Proteome Res. 2019;18(2):623–632. doi: 10.1021/acs.jproteome.8b00702 . 30450911 PMC6800166

[38] Lombardi F, Palumbo P, Augello FR, Cifone MG, Cinque B, & Giuliani M. Secretome of Adipose Tissue-Derived Stem Cells (ASCs) as a Novel Trend in Chronic Non-Healing Wounds: An Overview of Experimental In Vitro and In Vivo Studies and Methodological Variables. Int. J. Mol. Sci. 2019;20(15):3721. doi: 10.3390/ijms20153721 . 31366040 PMC6696601

[39] Peltzer J, Lund K, Goriot ME, Grosbot M, Lataillade JJ, Mauduit P, et al. Interferon-γ and Hypoxia Priming Have Limited Effect on the miRNA Landscape of Human Mesenchymal Stromal Cells-Derived Extracellular Vesicles. Front. Cell. Dev. Biol. 2020;8:581436. doi: 10.3389/fcell.2020.581436 . 33384991 PMC7769832

[40] Yoshida K, Nakashima A, Doi S, Ueno T, Okubo T, Kawano KI, et al. Serum-Free Medium Enhances the Immunosuppressive and Antifibrotic Abilities of Mesenchymal Stem Cells Utilized in Experimental Renal Fibrosis. Stem Cells Transl. Med. 2018;7(12):893–905. doi: 10.1002/sctm.17-0284 . 30269426 PMC6265641

[41] Zhao Y, Zhang M, Lu GL, Huang BX, Wang DW, Shao Y, et al. Hypoxic Preconditioning Enhances Cellular Viability and Pro-angiogenic Paracrine Activity: The Roles of VEGF-A and SDF-1a in Rat Adipose Stem Cells. Front. Cell. Dev. Biol. 2020;8:580131. doi: 10.3389/fcell.2020.580131 . 33330455 PMC7719676

[42] Ishiuchi N, Nakashima A, Doi S, Kanai R, Maeda S, Takahashi S, et al. Serum-free medium and hypoxic preconditioning synergistically enhance the therapeutic effects of mesenchymal stem cells on experimental renal fibrosis. Stem Cell Res Ther. 2021;12(1):472. doi: 10.1186/s13287-021-02548-7 . 34425892 PMC8381539

[43] Kakudo N, Morimoto N, Ma Y. & Kusumoto K. Differences between the Proliferative Effects of Human Platelet Lysate and Fetal Bovine Serum on Human Adipose-Derived Stem Cells. Cells. 2019;8(10):1218. doi: 10.3390/cells8101218 . 31597348 PMC6829610

[44] Shin J, Rhim J, Kwon Y, Choi SY, Shin S, Ha CW, et al. Comparative analysis of differentially secreted proteins in serum-free and serum-containing media by using BONCAT and pulsed SILAC. Sci. Rep. 2019;9(1):3096. doi: 10.1038/s41598-019-39650-z . 30816242 PMC6395664

[45] Lehrich BM, Liang Y. & Fiandaca MS. Foetal bovine serum influence on in vitro extracellular vesicle analyses. J. Extracell. Vesicles. 2021;10(3):e12061. doi: 10.1002/jev2.12061 33532042 PMC7830136

[46] Ludwig N, Whiteside TL & Reichert TE. Challenges in Exosome Isolation and Analysis in Health and Disease. Int J Mol Sci. 2019;20(19):4684. doi: 10.3390/ijms20194684 . 31546622 PMC6801453

[47] Zhang B, Wu Y, Mori M & Yoshimura K. Adipose-Derived Stem Cell Conditioned Medium and Wound Healing: A Systematic Review. Tissue Eng. Part B Rev 2022;28(4):830–847. doi: 10.1089/ten.TEB.2021.0100 . 34409890

[48] Gupta S, Rawat S, Krishnakumar V, Rao EP, & Mohanty S. Hypoxia preconditioning elicit differential response in tissue-specific MSCs via immunomodulation and exosomal secretion. Cell. Tissue Res. 2022;388(3):535–548. doi: 10.1007/s00441-022-03615-y . 35316374

[49] Garcia JP, Avila FR, Torres RA, Maita KC, Eldaly AS, Rinker BD, et al. Hypoxia-preconditioning of human adipose-derived stem cells enhances cellular proliferation and angiogenesis: A systematic review. J. Clin. Transl. Res. 2022;8(1):61–70. . 35187291 PMC8848748

[50] Cases-Perera O, Blanco-Elices C, Chato-Astrain J, Miranda-Fernández C, Campos F, Crespo PV, et al. Development of secretome-based strategies to improve cell culture protocols in tissue engineering. Scientific reports. 2022;12(1):10003. doi: 10.1038/s41598-022-14115-y . 35705659 PMC9200715

[51] Amable PR, Teixeira MV, Carias RB, Granjeiro JM, & Borojevic R. Protein synthesis and secretion in human mesenchymal cells derived from bone marrow, adipose tissue and Wharton’s jelly. Stem Cell Res Ther. 2014;5(2):53. doi: 10.1186/scrt442 . 24739658 PMC4055160

[52] An YH, Kim DH, Lee EJ, Lee D, Park MJ, Ko J, et al. High-Efficient Production of Adipose-Derived Stem Cell (ADSC) Secretome Through Maturation Process and Its Non-scarring Wound Healing Applications. Front Bioeng Biotechnol. 2021;9:681501. doi: 10.3389/fbioe.2021.681501 . 34222219 PMC8242583

[53] Liu L, Gao J, Yuan Y, Chang Q, Liao Y, & Lu F. Hypoxia preconditioned human adipose derived mesenchymal stem cells enhance angiogenic potential via secretion of increased VEGF and bFGF. Cell. Biol. Int. 2013;37(6):551–60. doi: 10.1002/cbin.10097 . 23505143

[54] Ong HT, Redmond SL, Marano RJ, Atlas MD, von Unge M, Aabel P, et al. Paracrine Activity from Adipose-Derived Stem Cells on In Vitro Wound Healing in Human Tympanic Membrane Keratinocytes. Stem Cells. Dev. 2017;26(6):405–418. doi: 10.1089/scd.2016.0204 . 28052725

[55] Follin B, Tratwal J, Haack-Sørensen M, Elberg JJ, Kastrup J & Ekblond A. Identical effects of VEGF and serum-deprivation on phenotype and function of adipose-derived stromal cells from healthy donors and patients with ischemic heart disease. J. Transl. Med. 2013;11:219. doi: 10.1186/1479-5876-11-219 . 24047149 PMC3852830

[56] Tratwal J, Mathiasen AB, Juhl M, Brorsen SK, Kastrup J, & Ekblond A. Influence of vascular endothelial growth factor stimulation and serum deprivation on gene activation patterns of human adipose tissue-derived stromal cells. Stem Cell. Res. Ther. 2015;6(1):62. doi: 10.1186/s13287-015-0062-9 . 25889587 PMC4431456

[57] Linero I & Chaparro O. Paracrine effect of mesenchymal stem cells derived from human adipose tissue in bone regeneration. PLoS One. 2014;9(9):e107001. doi: 10.1371/journal.pone.0107001 . 25198551 PMC4157844

[58] Heo SC, Jeon ES, Lee IH, Kim HS, Kim MB, & Kim JH. Tumor necrosis factor-α-activated human adipose tissue-derived mesenchymal stem cells accelerate cutaneous wound healing through paracrine mechanisms. J. Invest. Dermatol. 2011;131(7):1559–67. doi: 10.1038/jid.2011.64 . 21451545

[59] Li T, Ma H, Ma H, Ma Z, Qiang L, Yang Z, et al. Mussel-Inspired Nanostructures Potentiate the Immunomodulatory Properties and Angiogenesis of Mesenchymal Stem Cells. ACS Appl. Mater. Interfaces. 2019;11(19):17134–46. doi: 10.1021/acsami.8b22017 . 31008578

[60] Mehrabani M, Najafi M, Kamarul T, Mansouri K, Iranpour M, Nematollahi M, et al. Deferoxamine preconditioning to restore impaired HIF-1α-mediated angiogenic mechanisms in adipose-derived stem cells from STZ-induced type 1 diabetic rats. Cell. Prolif. 2015;48(5):532–49. doi: 10.1111/cpr.12209 . 26332145 PMC6495947

[61] Deng C, He Y, Feng J, Dong Z, Yao Y & Lu F. Conditioned medium from 3D culture system of stromal vascular fraction cells accelerates wound healing in diabetic rats. Regen. Med. 2019;14(10):925–937. doi: 10.2217/rme-2018-0083 . 31599183

[62] De Gregori C, Contador D, Díaz D, Cárcamo C, Santapau D, Lobos-Gonzalez L, et al. Human adipose-derived mesenchymal stem cell-conditioned medium ameliorates polyneuropathy and foot ulceration in diabetic BKS db/db mice. Stem Cell Res Ther. 2020;11(1):168. doi: 10.1186/s13287-020-01680-0 . 32357914 PMC7195803

[63] Liu J, Ren J, Su L, Cheng S, Zhou J, Ye X, et al. Human adipose tissue-derived stem cells inhibit the activity of keloid fibroblasts and fibrosis in a keloid model by paracrine signaling. Burns. 2018;44(2):370–385. doi: 10.1016/j.burns.2017.08.017 . 29029852

[64] Pu CM, Chen YC, Chen YC, Lee TL, Peng YS, Chen SH, et al. Interleukin-6 from Adipose-Derived Stem Cells Promotes Tissue Repair by the Increase of Cell Proliferation and Hair Follicles in Ischemia/Reperfusion-Treated Skin Flaps. Mediators Inflamm. 2019;2019:2343867. doi: 10.1155/2019/2343867 . 31814799 PMC6877947

[65] Xiao S, Deng Y, Mo X, Liu Z, Wang D, Deng C, et al. Promotion of Hair Growth by Conditioned Medium from Extracellular Matrix/Stromal Vascular Fraction Gel in C57BL/6 Mice. Stem Cells Int. 2020; 2020:9054514. doi: 10.1155/2020/9054514 . 32612663 PMC7306841

[66] Bodnar RJ, Satish L, Yates CC & Wells A. Pericytes: A newly recognized player in wound healing. Wound Repair. Regen. 2016;24(2):204–14. doi: 10.1111/wrr.12415 . 26969517 PMC5036393

[67] do Valle IB, Prazeres PHDM, Mesquita RA, Silva TA, de Castro Oliveira HM, Castro PR, et al. Photobiomodulation drives pericyte mobilization towards skin regeneration. Sci. Rep. 2020;10(1):19257. doi: 10.1038/s41598-020-76243-7 . 33159113 PMC7648092

[68] Birbrair A, Zhang T, Files DC, Mannava S, Smith T, Wang ZM, et al. Type-1 pericytes accumulate after tissue injury and produce collagen in an organ-dependent manner. Stem Cell Res Ther. 2014;5(6):122. doi: 10.1186/scrt512 . 25376879 PMC4445991

[69] Goss G, Rognoni E, Salameti V & Watt FM. Distinct Fibroblast Lineages Give Rise to NG2+ Pericyte Populations in Mouse Skin Development and Repair. Front. Cell. Dev. Biol. 2021; 9:675080. doi: 10.3389/fcell.2021.675080 . 34124060 PMC8194079

[70] Bogatcheva NV & Coleman ME. Conditioned Medium of Mesenchymal Stromal Cells: A New Class of Therapeutics. Biochemistry (Mosc). 2019;84(11):1375–1389. doi: 10.1134/S0006297919110129 . 31760924

[71] Vizoso FJ, Eiro N, Cid S, Schneider J, Perez-Fernandez R. Mesenchymal Stem Cell Secretome: Toward Cell-Free Therapeutic Strategies in Regenerative Medicine. Int J Mol Sci. 2017;18(9):1852. doi: 10.3390/ijms18091852 . 28841158 PMC5618501

[72] Abdel-Maguid EM, Awad SM, Hassan YS, El-Mokhtar MA, El-Deek HE & Mekkawy MM. Efficacy of stem cell-conditioned medium vs platelet-rich plasma as an adjuvant to ablative fractional CO2 laser resurfacing for atrophic post-acne scars: a split-face clinical trial. J. Dermatolog. Treat. 2021;32(2):242–249. doi: 10.1080/09546634.2019.1630701 . 31180258

