# Supplementary figures and images for: Secretome from human adipose-derived mesenchymal stem cells promotes blood vessel formation and pericyte coverage in experimental skin repair
Source: PLoS One. 2022 Dec 19;17(12):e0277863. doi: 10.1371/journal.pone.0277863 (PMC9762598; doi:10.1371/journal.pone.0277863)

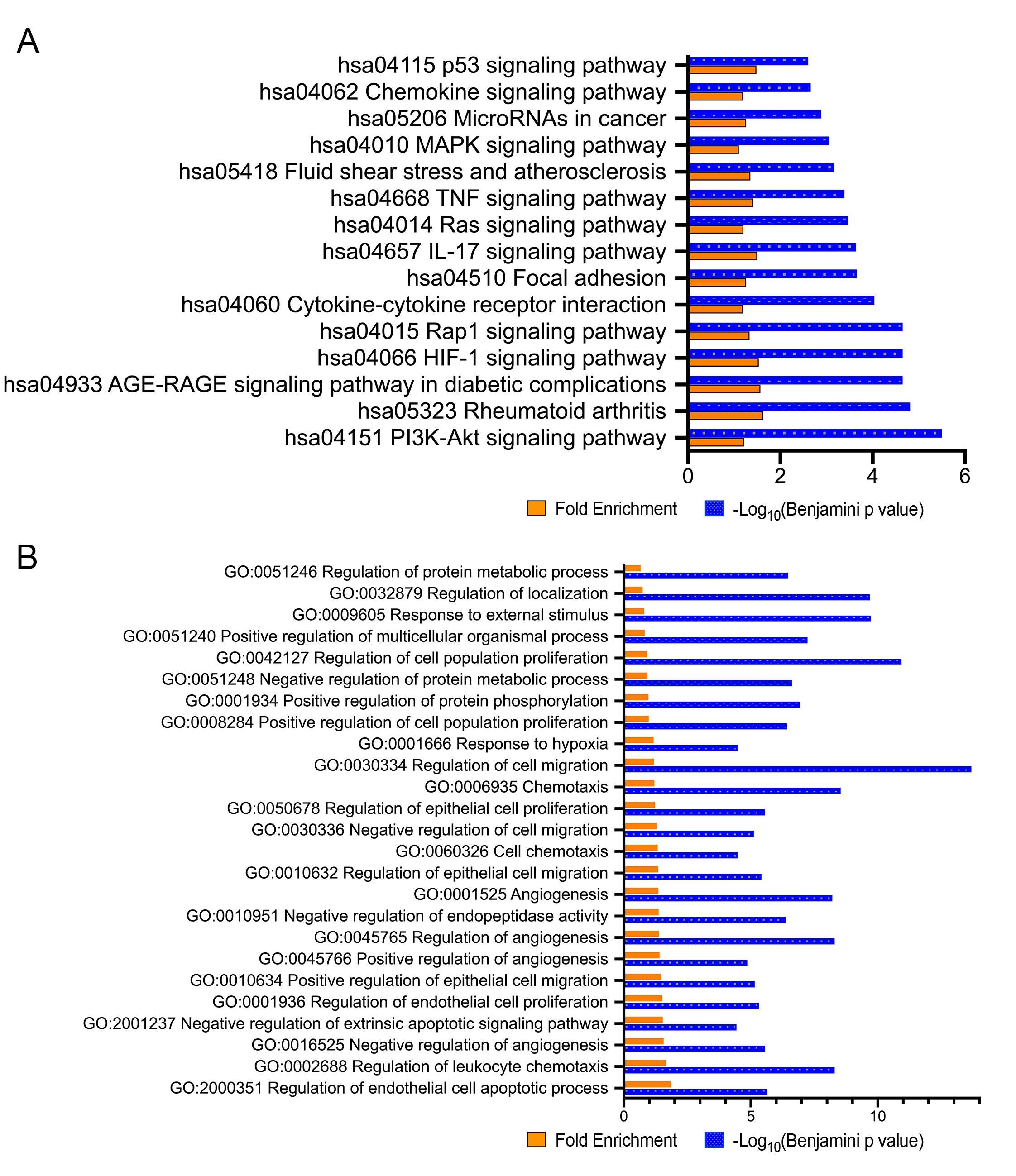

Supplement: S1 Fig — (A) KEGG enriched pathways of most representative trophic factors in hASC secretome ranked by p-value. − Log10 Benjamini p-value are reported as blue bar and Fold enrichment as orange bars. (B) Gene Ontology analyses of proteins more abundant in hASC conditioned medium ranked by Fold enrichment. The top 25 processes are shown. (TIFF) [file pone.0277863.s001.tiff]

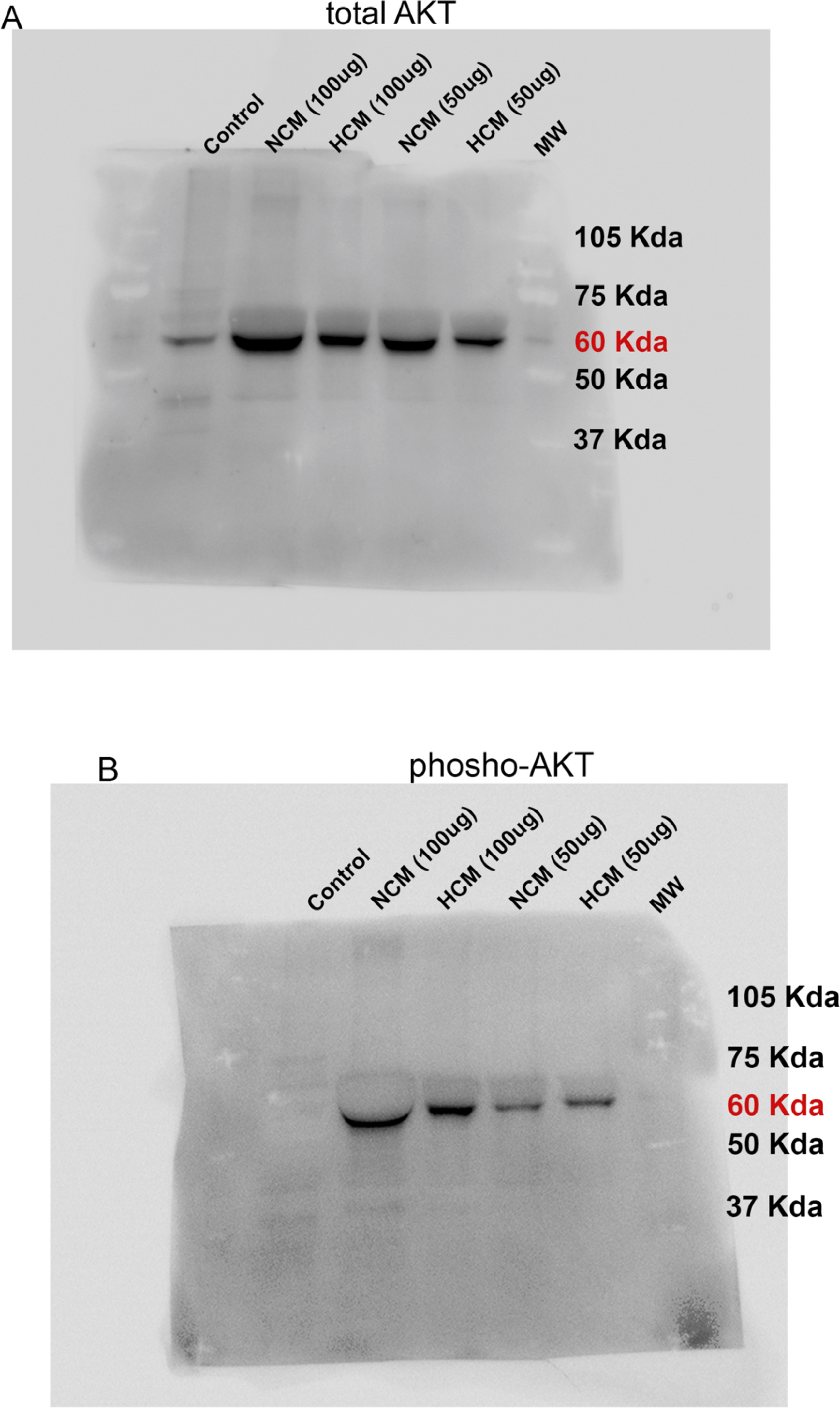

Supplement: S2 Fig — Representative Western blots for total-Akt (A) and P-AktSer473 (B) from HUVECs that were starved in 0.1%BSA EBM-2 and stimulated with control medium, NCM or HCM for 5 min. (JPG) [file pone.0277863.s002.jpg]

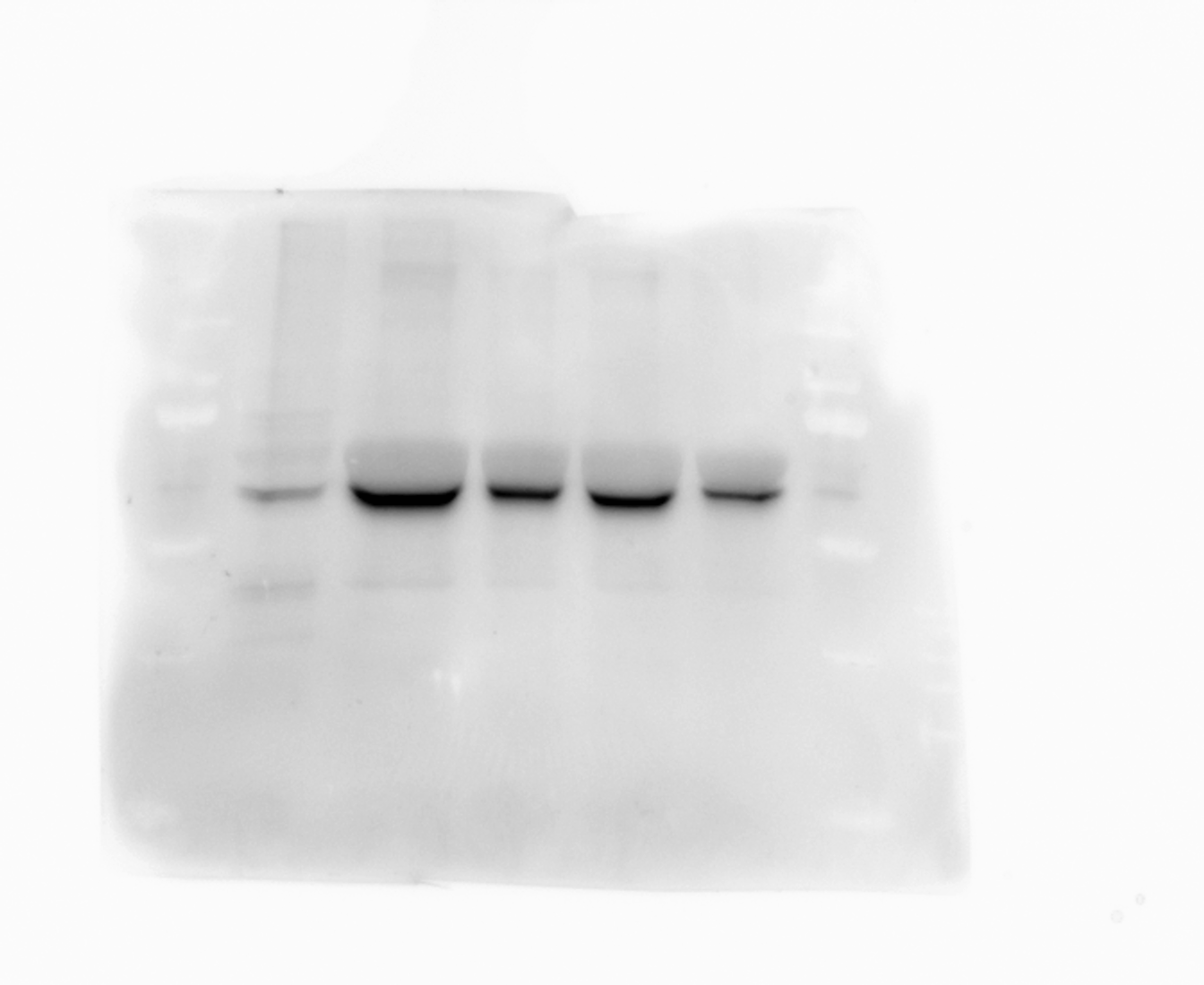

Supplement: S2 Raw images — (TIFF) [file pone.0277863.s004.tiff]
