# Supplementary material for: Secretome from human adipose-derived mesenchymal stem cells promotes blood vessel formation and pericyte coverage in experimental skin repair
Source: PLoS One. 2022 Dec 19;17(12):e0277863. doi: 10.1371/journal.pone.0277863 (PMC9762598; doi:10.1371/journal.pone.0277863)
Supplement: S1 Table — (DOCX) [file pone.0277863.s005.docx]

**S1 Table: Patient and tissue harvesting information**

| Identification | Ethnic group | Gender | Age | Source |
| --- | --- | --- | --- | --- |
| Patient 1 | Black | Female | 30 | abdominal liposuction |
| Patient 2 | White | Female | 45 | abdominal liposuction |
| Patient 3 | White | Female | 35 | abdominal liposuction |
| Patient 4 | White | Female | 27 | abdominal liposuction |
| Patient 5 | Black | Female | 38 | abdominal liposuction |
| Patient 6 | White | Female | 40 | abdominal liposuction |
| Patient 7 | White | Female | 25 | abdominal liposuction |
| Patient 8 | White | Female | 30 | abdominal liposuction |
